# Engineering, feasibility, and safety of force-controlled oropharyngeal swabs with a 3D-printed feedback system FCCSS (force controlled COVID-19 swab study) – a preliminary study 

**DOI:** 10.3205/dgkh000461

**Published:** 2024-02-21

**Authors:** Peter Melcher, Florian Metzner, Stefan Schleifenbaum, Toni Wendler, Paul Rahden, Corinna Pietsch, Pierre Hepp, Ralf Henkelmann

**Affiliations:** 1 Department of Orthopedics, Trauma and Plastic Surgery, University of Leipzig, Leipzig, Germany; 2 ZESBO – Center for Research on the Musculoskeletal System, University of Leipzig, Leipzig, Germany; 3 Department of Internal Medicine, Asklepios Hospital Nord-Heidberg, Hamburg, Germany; 4 Institute of Medical Microbiology and Virology, University of Leipzig, Leipzig, Germany

## Abstract

Errors in laboratory diagnostics of viral infections primarily occur during the preanalytical phase, which is especially observed in sample collection. Hitherto, no efforts have been made to optimize oropharyngeal smears. An accurate method to analyze the necessary conditions for a valid oropharyngeal smear test is required, especially to avoid false negative results, which can lead to promotion of the spread of viruses such as SARS-CoV-2.

In this study, a maximum-force failure analysis was performed on a swab, and the highest tolerable force was then measured on 20 healthy volunteers to obtain the dimensions of the possible force to be applied on a swab. Subsequently, a device which can validate and reproducibly indicate this force during swab collection was developed.

The study demonstrated that swabs generally fail at a maximum force of 5 N. Furthermore, an average force of 2.4±1.0 N was observed for the 20 volunteers. Lastly, this study described the development of a device which presents the selected force with a mean accuracy of 0.05 N (Force applied by Device 1: 0.46±0.05 N, Device 2: 1.55±0.11 N, Device 3: 2.57±0.18 N) and provides feedback via haptic and acoustic clicks as well as with a visual indicator. In the future, the swab will be analyzed for the presence of viral pathogens to determine its diagnostic performance corresponding to the force (German Clinical Trials Register Number 00024455).

## Abstract (de)

Fehler in der Labordiagnostik von Virusinfektionen treten vor allem in der präanalytischen Phase auf, was insbesondere bei der Probennahme zu beobachten ist. Bisher wurden keine Anstrengungen unternommen, um Oropharyngealabstriche zu optimieren. Eine genaue Methode zur Analyse der notwendigen Bedingungen für einen validen Oropharyngealabstrich ist daher erforderlich, insbesondere um falsch negative Ergebnisse zu vermeiden, die zur Förderung der Verbreitung von Viren wie SARS-CoV-2 führen können.

In dieser Studie wird eine Analyse der maximalen Kraft durchgeführt, die während eines oropharyngealen Abstrichs angewendet werden kann und die die maximale Versagenskraft eines üblichen Abstrichs bestimmt. Anschließend wird ein Gerät evaluiert, das diese Kraft während der Abstrichentnahme validieren und reproduzierbar anzeigen kann.

Die Studie zeigt, dass der untersuchte Abstrichtupfer bei einer maximalen Kraft von 5 N versagt. Außerdem wurde bei den 20 Probanden eine durchschnittliche tolerierte Kraft von 2,4±1,0 N festgestellt. Schließlich wird in dieser Studie die Entwicklung eines Geräts beschrieben, das die gewählte Kraft mit einer mittleren Genauigkeit von 0,05 N anzeigt (von Gerät 1 aufgebrachte Kraft: 0,46±0,05 N, Gerät 2: 1,55±0,11 N, Gerät 3: 2,57±0,18 N) und eine Rückmeldung über haptische und akustische Signale sowie eine visuelle Anzeige liefert. In Zukunft soll der Tupfer auf virale Erreger untersucht werden, um seine diagnostische Leistung entsprechend der Kraft zu bestimmen (Deutsches Register für klinische Studien, 00024455).

## Introduction

The pandemic spread of SARS-CoV-2 and the resulting COVID-19 disease places an enormous burden on both patients and healthcare systems globally. The main objective in tackling the pandemic is to limit the spread of infection. Thus, numerous tests have been conducted to detect this viral infection, primarily in respiratory specimens. Most of these tests rely on sample collection by swabs. The quality of the collected specimen has an enormous influence on the overall accuracy of the diagnostic procedure, in spite of the high sensitivity and specificity of polymerase chain reaction (PCR) tests [1], [2]. In the light of both the current SARS-CoV-2 pandemic and the well-known risks posed to humans by other respiratory viruses, the need for accurate respiratory swab procedures is not temporary and should be addressed [3].

Laboratory diagnostic procedures are divided into the following phases: test ordering (pre-preanalytical phase), diagnostic sample collection (preanalytical phase), sample analysis (analytical phase), reporting (postanalytical phase), and interpretation (postanalytical phase). However, most errors are observed to occur in the preanalytical phase, especially in the collection of the diagnostic samples with inappropriate or inadequate material (in terms of the quality or volume) [4], [5], [6]. Attempts have been made to counteract this phenomenon through various means, and standardized sample extractions have been performed in theory and in vivo. Furthermore, it is possible to produce a simulator for training purposes through 3D printing [7], [8]. However, limited research has been conducted to optimize and, more importantly, to standardize the sample collection process. An accurate method for the analysis of conditions necessary for a valid swab test is required to obtain quality control and valid results, and especially to avoid false negative results, which may promote the spread of SARS-CoV-2.

This study aims to present the development as well as the safety and feasibility of testing using a force-controlled swab device with the help of volunteers. It shows that a swab with a force of up to 4 N is safe, harmless, and feasible. Furthermore, this study also demonstrates the development of a device that reliably provides feedback to the system of a predefined force.

## Materials and methods

The study was performed in accordance with the principles of the Declaration of Helsinki. The study was approved by Ethics Committee of the Faculty of Medicine at Leipzig University (reference number: 057/20-ek). All the volunteers provided written consent to participate in the study after receiving the appropriate information. The volunteers were excluded if they had a tendency for bleeding, were taking anticoagulant medication, or had any anatomical deviations from the norm in the oropharynx.

Failure analysis of the swab was performed by applying an increasing force to five standard swabs which were vertically clamped in a universal testing machine (ZwickRoell GmbH & Co. KG, Z010 TE; Ulm, Germany). The force was applied by using a punch which was perpendicular to the swab. The force and the displacement were recorded to measure the critical buckling force and to obtain information on the occurrence of the breakage of the swab.

**Figure 1:**
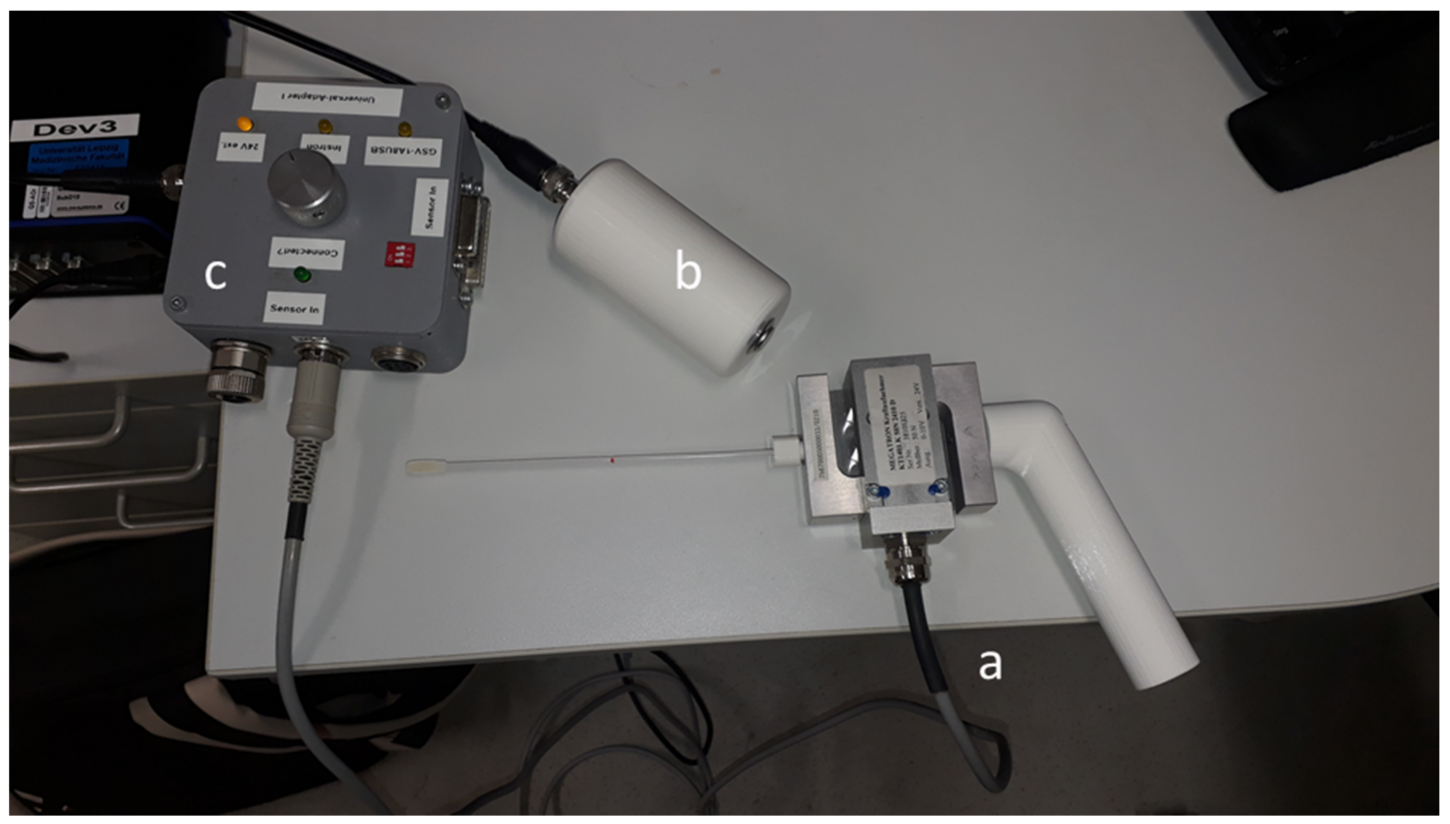
Device for force derivation of a swab; a=force transducer with swab, b=switch for test person to set marks in the force curve during measurement; c=control unit force transducer

For this study, 20 healthy volunteers were oropharyngeally swabbed thrice with a subjectively maximum tolerable force. The force-time curve and the highest applied force were derived using a force gauge attached to the swab. A 3D-printed handle and an adapter were mounted on a force transducer (S-Beam Force Transducer KT1401 50N, MEGATRON Elektronik GmbH & Co. KG, Putzbrunn/München, Germany). The swab was held in the adapter with the help of aclamp. Data processing was performed using the LabVIEW routine. During measuring, feedback was collected from the patient via a hand-held button which they pressed when subjective maximum was reached (Figure 1 (Fig. 1)). 

This point was automatically marked during the measurement and was fed back to the examiner using a visual and acoustic signal, after which the examiner immediately released the force, ending the measurement recording. Neither the subject nor the examiner received any feedback about the applied forces. The subjects rated the symptoms/pain using a visual analogue scale (VAS) between 0 and 10 (least to most severe), immediately, after 15 min, and after 1 h. Furthermore, they were asked to indicate when the pain/symptoms were no longer present (in min) or to describe them in a free text field.

**Figure 2:**
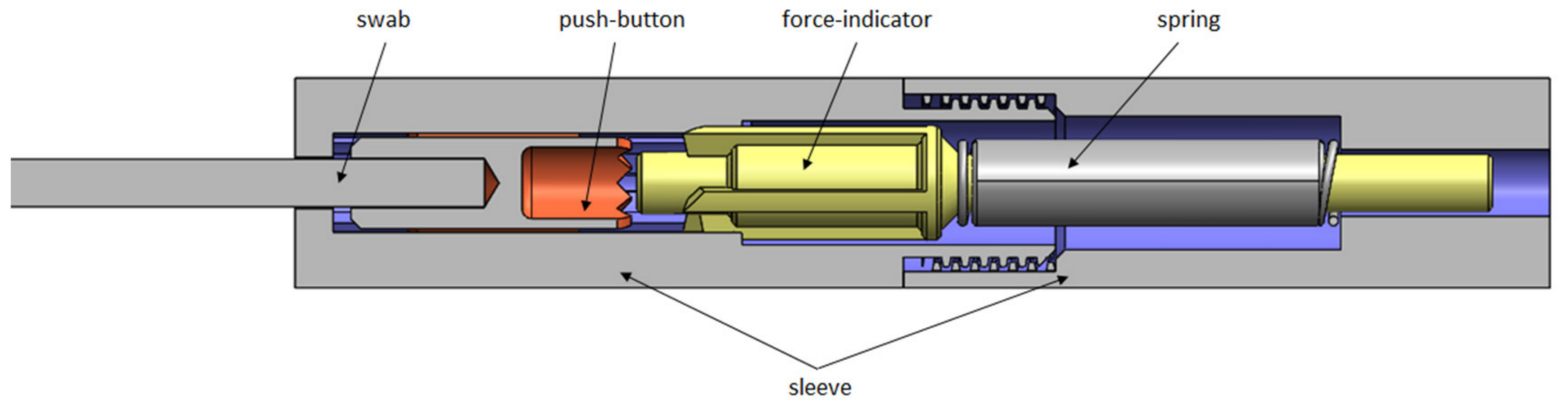
Sketch of the device developed for maintaining force on the swab

A device was developed to maintain a predefined force on the swab. It consists of a rotating mechanism similar to a ballpoint pen, which uses the internal spring for force control, as shown in Figure 2 (Fig. 2). The swab is attached to the “push-button” of the ballpoint pen. The geometry and the spring are designed such that the inner rotating mechanism switches precisely at the desired force with the characteristic click-sound, which provides the user with the haptic and acoustic feedback (Figure 2 (Fig. 2)).

Additionally, if the desired force is exceeded, the rotational part emerges from the back end and provides the user with optical feedback. The device was 3D-printed from rigid plastic (BioMed Amber) using stereolithography (Form 3 B, Formlabs, Somerville, Massachusetts, USA). The biocompatible material is certified according to the Medical Device Regulation EN ISO 13485. The compression springs for the device were selected so that three resultant forces were obtained using the mean tolerable swab force. The device was tested using the previously mentioned force measuring setup for functional validation (Figure 1 (Fig. 1)). The force transducer was mounted on a table using a clamp. The swab mounted in the device was manually pushed against the transducer 20 times. The force was released immediately after the “click” as intended, and the maximum force was recorded. Two devices were manufactured and labeled for each force configuration (S-Soft, M-Medium, H-Hard).

The data were characterized using standard statistics: mean value (standard deviation) for continuous data and number (percent) for categorical data. The data were compared using either the t-test, chi-squared test, or the Mann-Whitney U-test, depending on the variables being compared. All tests were two-sided with a significance level of α=0.05. The analyses were performed using IBM SPSS Statistics version 26 software. 

## Results

**Figure 3:**
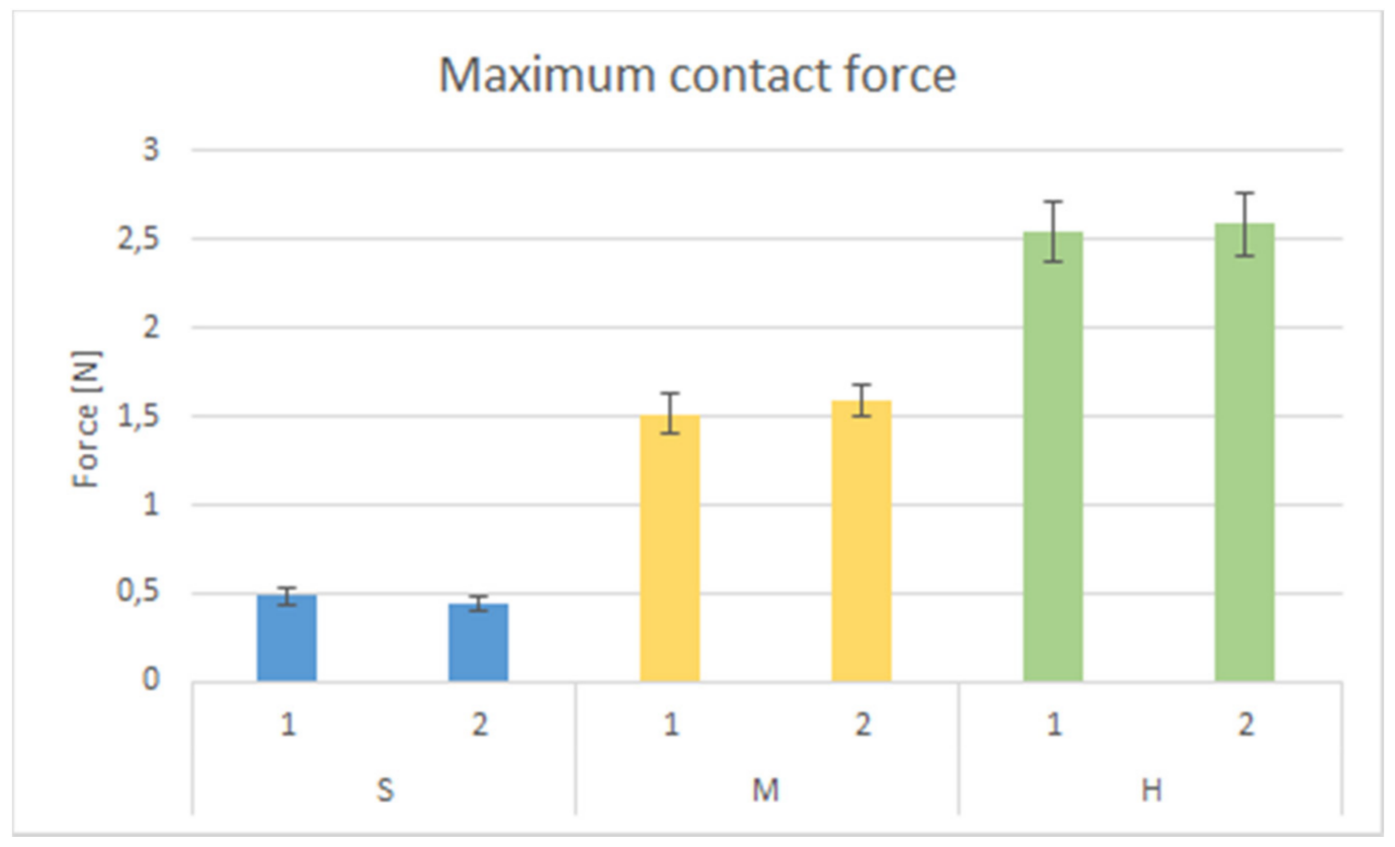
Results of validation analysis. Two devices were tested 20 times for each type (S: soft, M: Medium, H: Hard).

A maximum load of 5.2±0.1 N was recorded in the failure analysis of the swab. The swab buckled at the maximum force following an initial linear elastic region. With a higher displacement, the force remained relatively constant until the swab slipped horizontally at displacements of at least 20 mm. None of the swabs failed during testing. (Fig. 3)

Thirteen male and seven female volunteers with an average age of 30.7±4.1 years (range 24–40 years) were swabbed according to the study protocol. The measured force during swabbing was 2.4±1.0 N (range 0.6–4.4 N). The mean maximum pain measured directly after swabbing was 4.7±1.7 (range 2–7). 15 min after swabbing, the volunteers still reported discomfort or pain of 0.2±0.4 (range 0–1). One hour after swabbing, none of the volunteers reported any pain or discomfort. The duration until the complete disappearance of the discomfort or pain was recorded at 8.7±7.5 min (range: 1–30 min). None of the volunteers reported any symptoms during the monitoring period, other than discomfort or pain from the swab at the swab site. 

Based on the previously mentioned mean tolerable force of 2.5 N, the devices are required to maintain the following forces: S=0.5 N, M=1.5 N, H=2.5 N. Three different springs were used to achieve these forces, (Article Number: 0X-RDF1189; 0C0180-0141000S; 0X-DF1281, Febrotec GmbH, Halver, Germany). Figure 3 (Fig. 3) shows the maximum reaction forces measured during the validation analysis. 

The maximum contact forces of each device type (S, M, and H) are 0.46±0.05 N, 1.55±0.11 N, and 2.57±0.18 N, respectively, which presents a mean accuracy of 0.05 N for the device.

## Discussion

This study aims to determine the maximum tolerable force for an oropharyngeal swab. Although nasopharyngeal swabs are often recommended for the detection of SARS-CoV-2 from the upper respiratory tract [1], oropharyngeal swab collection is more tolerable for most patients, with comparable or just slightly lower diagnostic sensitivity [9].

The average maximum tolerable force of a swab was significantly lower than the force at which the swab breaks. Furthermore, there were no complications even with a maximum tolerable force, and the discomfort or pain was no longer present after approximately 9 min. Based on these findings, it can be stated that it is safe and acceptable to perform an oropharyngeal swab on a patient with a force of 2.5 N. 

The device developed in this study can be equipped with an appropriate force spring to ensure that a force-controlled swab can be used reproducibly and reliably with a predefined force. 

It should be borne in mind that this study primarily focusses on the improvement and standardization of swabs collected by healthcare workers. Self-collected swabs are known to be of inferior quality, and improvement of those would certainly need another approach than the swabbing presented here.

Altogether, force-controlled swabbing appears to be promising for optimization of the error-prone pre-analytic stage. The feasibility and effect of different forces on the swab as well as on diagnostic test quality and accuracy will be analyzed in the subsequent prospective study (DRKS00024455).

## Notes

### Competing interests

The authors declare that they have no competing interests.

### Authorsip

Peter Melcher and Florian Metzner contributed equally.

### Availability of data and material

Further data of the study are available on request from the corresponding author.

### Author’s ORCID

- Peter Melcher: 0000-0002-4747-8488
